# Phylogenetic Analysis of Hemagglutinin Genes of H9N2 Avian Influenza Viruses Isolated from Chickens in Shandong, China, between 1998 and 2013

**DOI:** 10.1155/2015/267520

**Published:** 2015-11-02

**Authors:** Yuxin Zhao, Song Li, Yufa Zhou, Wengang Song, Yujing Tang, Quanhai Pang, Zengmin Miao

**Affiliations:** ^1^College of Life Sciences, Taishan Medical University, Tai'an 271000, China; ^2^College of Basic Medicine, Taishan Medical University, Tai'an 271000, China; ^3^College of Animal Science and Technology, Shanxi Agricultural University, Taigu 030800, China; ^4^Animal Disease Control Center, Animal Husbandry Bureau of Daiyue, Tai'an 271000, China

## Abstract

Since H9N2 avian influenza virus (AIV) was first isolated in Guangdong province of China, the virus has been circulating in chicken flocks in mainland China. However, a systematic phylogenetic analysis of H9N2 AIV from chickens in Shandong of China has not been conducted. Based on hemagglutinin (HA) gene sequences of H9N2 AIVs isolated from chickens in Shandong of China between 1998 and 2013, genetic evolution of 35 HA gene sequences was systematically analyzed in this study. Our findings showed that the majority of H9N2 AIVs (21 out of 35) belonged to the lineage h9.4.2.5. Most of isolates (33 out of 35) had a PSRSSR↓GLF motif in HA cleavage site. Importantly, 29 out of these 35 isolates had an amino acid exchange (Q226L) in the receptor-binding site. The substitution showed that H9N2 AIVs had the potential affinity to bind to human-like receptor. The currently prevalent H9N2 AIVs in Shandong belonged to the lineage h9.4.2.5 which are different from the vaccine strain SS/94 clade h9.4.2.3. Therefore, the long-term surveillance of H9N2 AIVs is of significance to combat the possible H9N2 AIV outbreaks.

## 1. Introduction

Since H9N2 subtype avian influenza virus (AIV) was first isolated in Guangdong province of China in 1994, this subtype influenza virus has quickly spread to other areas of China and become prevalent in poultry [1–3]. H9N2 AIV infections in chicken flocks have led to massive economic losses due to egg production decline or high mortality related with coinfection with other pathogens [4–6].

Although there is no evidence for human-to-human transmission of H9N2 AIV, several human infections with H9N2 AIV have been reported [7–11]. It is noteworthy that the number of humans infected by H9N2 AIV in serological surveillance, especially poultry workers, was much higher than that of the confirmed cases [12, 13]. H9N2 AIV is able to reassort with other subtypes of influenza virus, including H6N1, H6N2, H5N1, and H7N9. The increased risk of animal-to-human spread with avian viruses that have the H9N2 internal genes has posed a greater pandemic threat [14–16].

Since the late 1990s, vaccination has been carried out in China to prevent and control H9N2 AIV infection in chicken flocks. Commercial vaccines did not provide complete protection for the endemic H9N2 AIVs in China. Up to the present time, H9N2 virus has regularly been found in vaccinated chickens in Shandong of China [1, 17, 18]. Therefore, this study was designed to perform a comprehensive phylogenetic analysis of H9N2 AIVs from chickens in Shandong of China to provide an important guidance for the effective control of H9N2 AIV infection. 

## 2. Materials and Methods

### 2.1. Viruses

A total of 35 HA gene sequences of H9N2 AIVs isolated from chickens in Shandong of China between 1998 and 2013 were obtained from the National Center for Biotechnology Information (NCBI) Influenza Viruses Resource (http://www.ncbi.nlm.nih.gov/genomes/FLU/FLU.html) (Table 1).

### 2.2. Phylogenetic Analysis

Multiple sequence alignment was conducted using ClustalW [19]. The phylogenetic tree was constructed using the neighbor-joining method with 1000 bootstrap replicates using MEGA 5.05 [20].

### 2.3. Antigenic Analysis

Antigenic analysis of H9N2 AIV was conducted using hemagglutination inhibition (HI) to assess antigenic relationship between the emerging H9N2 AIVs and the vaccine strain SS/94 [21]. According to the previously published work [22], 6-week-old specific pathogen-free (SPF) chickens were used to produce polyclonal antibodies against 7 H9N2 AIVs, including 6 isolates between 2012 and 2013, and the vaccine strain SS/94.

## 3. Results

### 3.1. Homology Analysis

The coding sequences of HA gene of 35 H9N2 AIVs in this study contained 1,683 nucleotides. HA gene nucleotide sequences and deduced amino acid (AA) sequence identities among the 35 tested strains ranged from 84.0 to 100.0% and 89.1 to 100.0%, respectively. Compared with three vaccine strains, the homologies of HA nucleotide sequences and AA sequence entities of the 35 H9N2 AIVs were 84.0–93.0% and 83.6–92.2%, respectively (Table 2).

### 3.2. Phylogenetic Analysis

**Figure 1:**
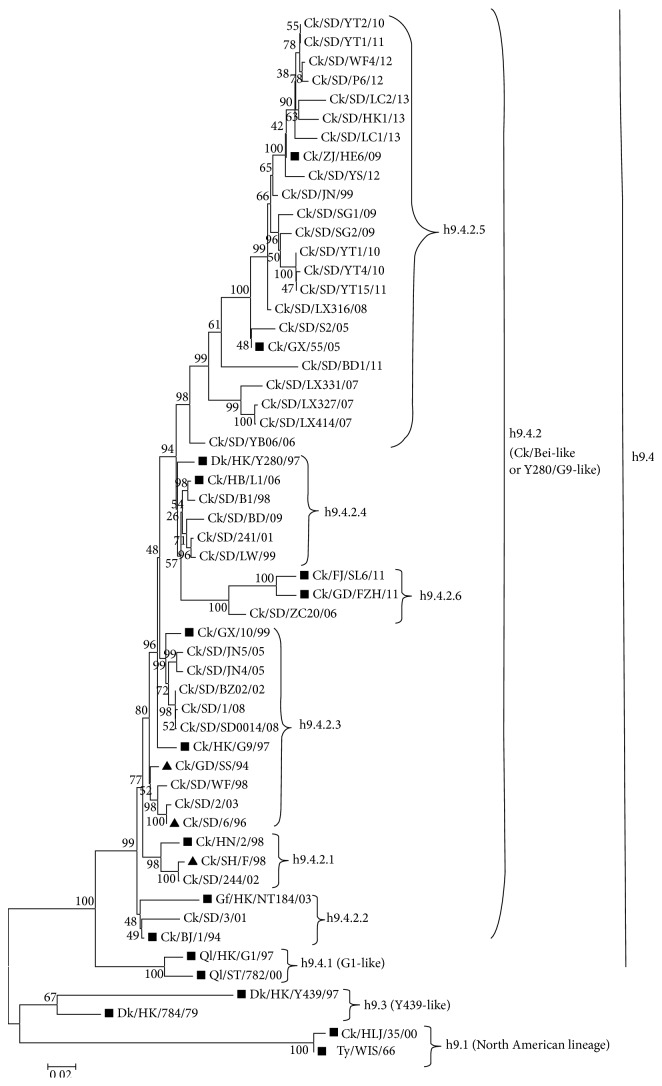
Phylogenetic tree of HA genes of H9N2 AIVs used in this study. The black squares indicate the representative strains of distinct H9 lineages. The black triangles indicate vaccine strains.

All of 35 H9N2 AIVs belonged to the lineage h9.4.2 represented by Y280 or BJ194. The lineage h9.4.2 is divided into 6 tertiary levels: h9.4.2.1, h9.4.2.2, h9.4.2.3, h9.4.2.4, h9.4.2.5, and h9.4.2.6 [23]. The majority of H9N2 AIVs (21 out of 35) belonged to the lineage h9.4.2.5 represented by strains Ck/ZJ/HE6/09 and Ck/GX/55/05 [24]. All of 12 H9N2 AIVs isolated between 2010 and 2013 belonged to the lineage h9.4.2.5. Vaccine strains SS/94 and 6/96 belonged to the linage h9.4.2.3, and vaccine strain F/98 was clustered in the lineage h9.4.2.1 (Figure 1).

### 3.3. Important Site Analysis of Deduced AA Sequences

The cleavage sites of precursor HA protein of H9N2 AIVs showed two different motifs in this study. 33 H9N2 AIVs isolated between 1999 and 2013 had a PSRSSR↓GLF motif, which can meet the characteristic of low-pathogenic avian influenza virus (Table 3).

AA residues at receptor-binding site of HA protein were conserved among the 35 H9N2 AIVs. Only one substitution was found in the position A190V(T) (H3 numbering is used throughout the paper) (Table 3). On the right edge of receptor-binding pocket, AA sequences were conserved and no substitution was found in this study. AA residues on the left edge of receptor-binding pocket had a substitution Q227L (M), and most isolates (4 out of 6) between 2012 and 2013 had M at position 227. 29 out of 35 H9N2 AIVs had leucine (L) at position 226, including all of the isolates between 2005 and 2013 (Table 3).

### 3.4. Antigenic Analysis

HI test was used to evaluate protection efficacy of commercial vaccine strain SS/94 widely used in Shandong against 6 H9N2 AIVs isolated in Shandong between 2012 and 2013. The results showed that antisera against 6 H9N2 AIVs (Ck/SD/YS/12, Ck/SD/WF4/12, Ck/SD/P6/12, Ck/SD/HK1/13, Ck/SD/LC1/13, and Ck/SD/LC2/13) reacted well with the emerging H9N2 AIVs (HI titer ≥ 1280), but none of antisera against the emerging H9N2 AIVs reacted well with the vaccine strain SS/94 (HI titer ≤ 320) (Table 4).

## 4. Discussion

Vaccination is an effective way to prevent and control the spread of H9N2 AIVs, but the current vaccine used in Shandong is still prepared from the isolates in the early 1990s, and their protective efficacy has been decreasing [3, 18]. HI test in this study showed that commercial vaccine strain SS/94 has not provided robust protection against the emerging H9N2 AIVs isolated in Shandong, China.

Previous investigation about phylogenetic analysis of HA genes of H9N2 AIVs isolated in China between 2008 and 2011 demonstrated that the lineage h9.4.2.5, as an emerging lineage, has become one of the dominant clades [22, 25]. Similarly, our findings showed that the lineage h9.4.2.5 was predominant between 2009 and 2013 in Shandong. The vaccine strain SS/94 belonged to the distinct lineage h9.4.2.3. Antigenic analysis also showed that the emerging viruses between 2012 and 2013 were antigenically different from the vaccine strain SS/94. Given the above results, it is of utmost importance to select novel candidate vaccine strains to combat the emerging H9N2 AIVs in Shandong of China.

On the left edge of receptor-binding pocket, most isolates (4 out of 6) between 2012 and 2013 had methionine (M) at position 227. But the vaccine strain SS/94 had glutamine (Q) at the same position. 33 H9N2 AIVs isolated between 1999 and 2013 had a PSRSSR↓GLF motif in HA cleavage site; the vaccine strain SS/94 had a PAGSSR↓GLF motif. Additionally, 29 out of 35 H9N2 AIVs had leucine (L) at position 226, including all of the isolates between 2005 and 2013. The substitution indicated that the emerging H9N2 AIVs can preferentially bind to NeuAca2,6-Gal linkage and may have higher virulence [3, 26].

Our findings revealed that the currently prevalent H9N2 AIVs in Shandong belonged to the lineage h9.4.2.5 which are different from the vaccine strain SS/94 clade h9.4.2.3. Furthermore, 82.9% (29/35) of H9N2 AIVs contained L at position 226, indicating that these AIVs could cross species barrier to infect humans.

## 5. Conclusion

Taken together, genetic disparity between the emerging H9N2 AIVs and the current vaccine isolates should be taken into consideration when a control policy is established. In addition, the long-term surveillance of H9N2 AIVs is of significance to combat the possible H9N2 AIV outbreaks in chicken flocks.

## Figures and Tables

**Table 1:** HA genes of 35 H9N2 AIVs isolated from Shandong of China from 1998 to 2013.

Strains	Abbreviation	Date of isolation	GenBank accession number
A/chicken/Shandong/WF/1998(H9N2)	Ck/SD/WF/98	1998	JF795136
A/chicken/Shandong/B1/1998(H9N2)	Ck/SD/B1/98	1998	EU573939
A/chicken/Shandong/JN/1999(H9N2)	Ck/SD/JN/99	1999	HM773437
A/chicken/Shandong/LW/1999(H9N2)	Ck/SD/LW/99	1999	HM773438
A/chicken/Shandong/241/2001(H9N2)	Ck/SD/241/01	2001	KF746827
A/chicken/Shandong/3/2001(H9N2)	Ck/SD/3/01	2001	KF313566
A/chicken/Shandong/244/2002(H9N2)	Ck/SD/244/02	2002	KF746810
A/chicken/Shandong/BZ02/2002(H9N2)	Ck/SD/BZ02/02	2002	JQ710462
A/chicken/Shandong/2/2003(H9N2)	Ck/SD/2/03	2003	FJ190117
A/chicken/Shandong/S2/2005(H9N2)	Ck/SD/S2/05	2005	HM773440
A/chicken/Shandong/JN5/2005(H9N2)	Ck/SD/JN5/05	2005	HM773436
A/chicken/Shandong/JN4/2005(H9N2)	Ck/SD/JN4/05	2005	HM773435
A/chicken/Shandong/ZC820/2006(H9N2)	Ck/SD/ZC820/06	2006	FJ190135
A/chicken/Shandong/YB06/2006(H9N2)	Ck/SD/YB06/06	2006	JQ710463
A/chicken/Shandong/LX414/2007(H9N2)	Ck/SD/LX414/07	2007	FJ190141
A/chicken/Shandong/LX331/2007(H9N2)	Ck/SD/LX331/07	2007	FJ190140
A/chicken/Shandong/LX327/2007(H9N2)	Ck/SD/LX327/07	2007	FJ190139
A/chicken/Shandong/SD0014/2008(H9N2)	Ck/SD/SD0014/08	2008	HQ398361
A/chicken/Shandong/1/2008(H9N2)	Ck/SD/1/08	2008	HQ326722
A/chicken/Shandong/LX316/2008(H9N2)	Ck/SD/LX316/08	2008	FJ190138
A/chicken/Shandong/BD/2009(H9N2)	Ck/SD/BD/09	2009	HM773434
A/chicken/Shandong/SG2/2009(H9N2)	Ck/SD/SG2/09	2009	HM751194
A/chicken/Shandong/SG1/2009(H9N2)	Ck/SD/SG1/09	2009	HM751186
A/chicken/Shandong/YT1/2010(H9N2)	Ck/SD/YT1/10	2010	KJ419948
A/chicken/Shandong/YT2/2010(H9N2)	Ck/SD/YT2/10	2010	KJ419946
A/chicken/Shandong/YT4/2010(H9N2)	Ck/SD/YT4/10	2010	KJ419943
A/chicken/Shandong/BD1/2011(H9N2)	Ck/SD/BD1/11	2011	KJ419954
A/chicken/Shandong/YT15/2011(H9N2)	Ck/SD/YT15/11	2011	KJ419953
A/chicken/Shandong/YT1/2011(H9N2)	Ck/SD/YT1/11	2011	KJ419947
A/chicken/Shandong/YS/2012(H9N2)	Ck/SD/YS/12	2012	KC879302
A/chicken/Shandong/WF4/2012(H9N2)	Ck/SD/WF4/12	2012	JX448768
A/chicken/Shandong/P6/2012(H9N2)	Ck/SD/P6/12	2012	JX448767
A/chicken/Shandong/HK1/2013(H9N2)	Ck/SD/HK1/13	2013	KJ419952
A/chicken/Shandong/LC1/2013(H9N2)	Ck/SD/LC1/13	2013	KJ419951
A/chicken/Shandong/LC2/2013(H9N2)	Ck/SD/LC2/13	2013	KJ419950

**Table 2:** Similarity analysis of HA nucleotide sequence and deduced amino acid sequence of H9N2 AIVs and vaccine strains.

	Virus	Nucleotide sequence	Amino acid sequence
Vaccine strains	35 H9N2 AIVs in this study	84.0–100.0%	89.1–100.0%
	A/chicken/Guangdong/SS/1994	86.2–92.1%	83.6–87.5%
	A/chicken/Shandong/6/1996	84.5–91.4%	86.7–89.2%
	A/chicken/Shanghai/F/1998	84.0–93.0%	87.7–92.2%

**Table 3:** Amino acid sequence at the cleavage site and receptor-binding site of HA.

Strain abbreviation	Left edge	Receptor-binding sites (RBS)							Right edge	Cleavage site
SD/6/96	224–229	98	153	155	183	190	194	195	134–138	
	NGQQGR	P	W	T	N	A	L	Y	GTSKA	PARSSR↓GLF
SD/WF/98	+	+	+	+	+	T	+	+	+	+
SD/B1/98	+	+	+	+	+	T	+	+	+	+
SD/JN/99	NGLQGR	+	+	+	+	+	+	+	+	PSRSSR↓GLF
SD/LW/99	+	+	+	+	+	V	+	+	+	+
SD/241/01	+	+	+	+	+	V	+	+	+	+
SD/3/01	NGMQGR	+	+	+	+	V	+	+	+	+
SD/244/02	+	+	+	+	+	+	+	+	+	+
SD/BZ02/02	+	+	+	+	+	T	+	+	+	+
SD/2/03	+	+	+	+	+	+	+	+	+	+
SD/S2/05	NGLQGR	+	+	+	+	+	+	+	+	+
SD/JN5/05	+	+	+	+	+	V	+	+	+	+
SD/JN4/05	+	+	+	+	+	V	+	+	+	+
SD/ZC820/06	+	+	+	+	+	+	+	+	+	+
SD/YB06/06	+	+	+	+	+	T	+	+	+	+
SD/LX414/07	+	+	+	+	+	V	+	+	+	+
SD/LX331/07	+	+	+	+	+	+	+	+	+	+
SD/LX327/07	+	+	+	+	+	T	+	+	+	+
SD/SD0014/08	+	+	+	+	+	T	+	+	+	+
SD/1/08	+	+	+	+	+	V	+	+	+	+
SD/LX316/08	+	+	+	+	+	V	+	+	+	+
SD/BD/09	+	+	+	+	+	V	+	+	+	+
SD/SG2/09	+	+	+	+	+	T	+	+	+	+
SD/SG1/09	+	+	+	+	+	T	+	+	+	+
SD/YT1/10	+	+	+	+	+	+	+	+	+	+
SD/YT2/10	+	+	+	+	+	+	+	+	+	+
SD/YT4/10	+	+	+	+	+	+	+	+	+	+
SD/BD1/11	+	+	+	+	+	V	+	+	+	+
SD/YT15/11	+	+	+	+	+	+	+	+	+	+
SD/YT1/11	NGLMGR	+	+	+	+	V	+	+	+	+
SD/YS/12	NGLQGR	+	+	+	+	V	+	+	+	+
SD/WF4/12	NGLMGR	+	+	+	+	V	+	+	+	+
SD/P6/12	+	+	+	+	+	V	+	+	+	+
SD/HK1/13	+	+	+	+	+	V	+	+	+	+
SD/LC1/13	+	+	+	+	+	+	+	+	+	+
SD/LC2/13	+	+	+	+	+	T	+	+	+	+

*Note*. Plus signs (+) indicate identical site.

**Table 4:** HI titers of the emerging H9N2 AIVs isolated in 2012 and 2013.

Antigen	Antisera^A^						
	SD/YS/12	SD/WF4/12	SD/P6/12	SD/HK1/13	SD/LC1/13	SD/LC2/13	SS/94
SD/YS/12	10240^B^	2560	1280	1280	1280	1280	320
SD/WF4/12	2560	**10240**	1280	2560	1280	2560	160
SD/P6/12	2560	5120	**2560**	1280	5120	2560	320
SD/HK1/13	5120	10240	1280	**10240**	1280	2560	320
SD/LC1/13	2560	5120	5120	1280	**5120**	2560	160
SD/LC2/13	1280	2560	1280	2560	1280	**10240**	160
SS/94	320	320	40	160	320	40	**1280**

*Notes*. ^A^Antisera were tenfold diluted; ^B^homologous tiers were marked in bold.
